# Supplementary material for: Global spatiotemporal synchronizing structures of spontaneous neural activities in different cell types
Source: Nat Commun. 2024 Apr 3;15:2884. doi: 10.1038/s41467-024-46975-5 (PMC10991327; doi:10.1038/s41467-024-46975-5)
Supplement: Supplementary file 1 — Supplementary Information [file 41467_2024_46975_MOESM1_ESM.pdf]

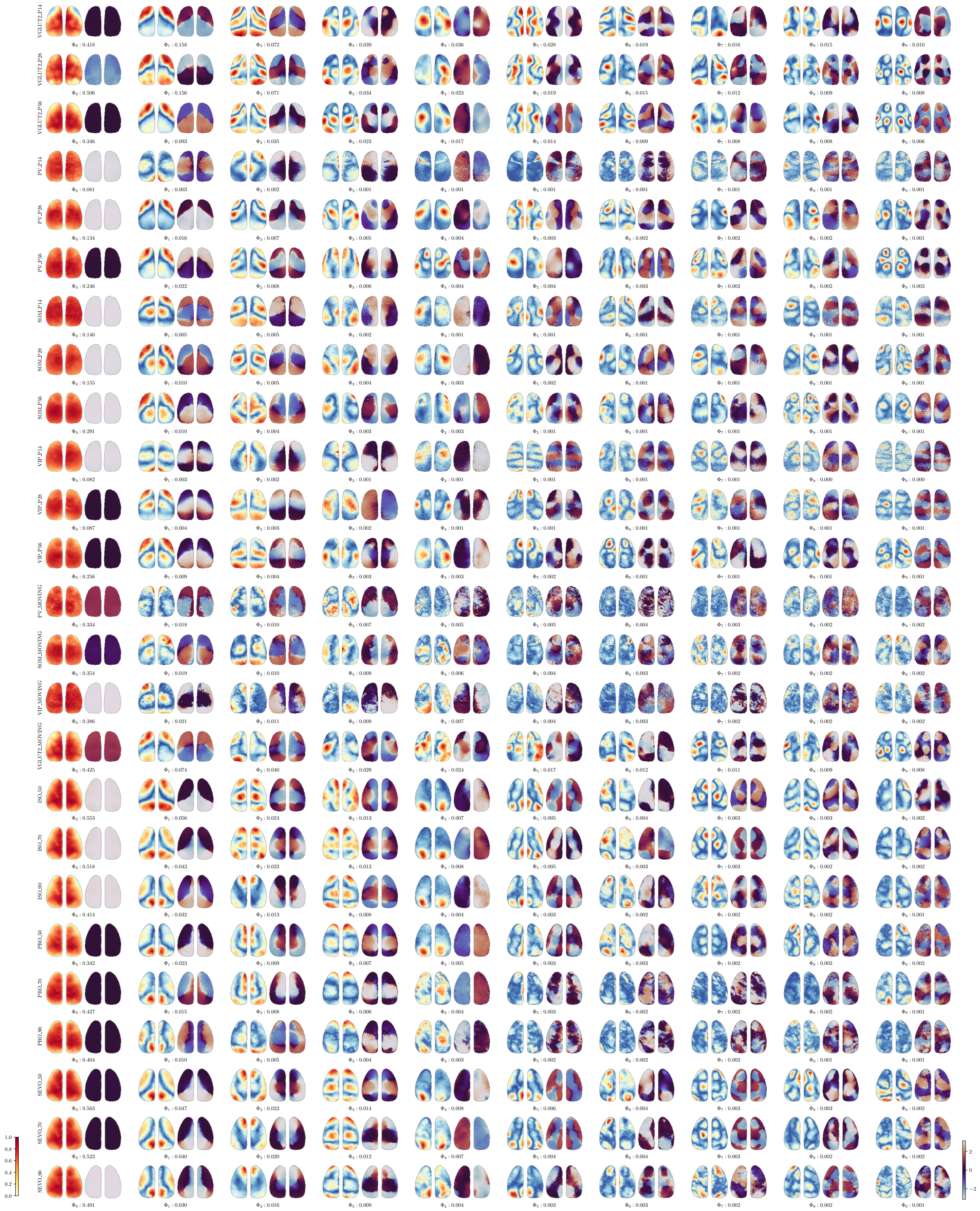

Supplementary Fig. 1: Spatial distribution of strongest 10 waves at each state. The left panel of each figure shows the spatial distribution of wave intensity, and the right panel shows the spatial distribution of wave phase. The numbers at bottom indicate explained variance of each wave (without GSR). The relation shape between explained variance with GSR (s_i^{GSR}, showed in Fig 5 and 7) and without GSR s_i. $s_i^{GSR} = s_i/(1 - s_0)$, and relative explained variance are $s_i^{reletive} = s_i/\sum_i s_i$.

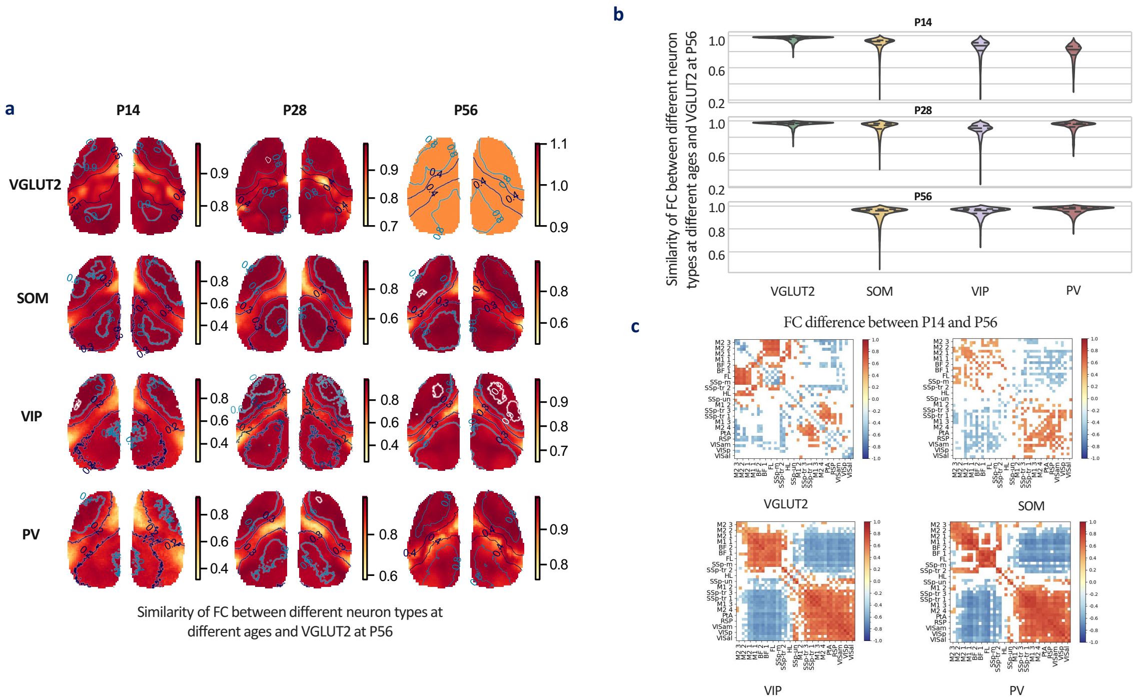

Supplementary Fig. 2: Similarity between FC and standing/traveling waves. (a) The spatial distribution of similarity between the RSFC of different neuron types and that of VGLUT2 at P56 (RSFC similarity), measured by Pearson coefficient of RSFC at each pixel. The contour illustrates the spatial distribution of Φ_1. (b) The violine plot of RSFC similarity. (c) Changes of seed-seed FC post GSR in four types of neurons between P14 and P56, with diagonal entries representing the short-range FC changes ($p < 0.05$, Fisher's z-transformed t-tests, after FDR correction with threshold of 0.05).

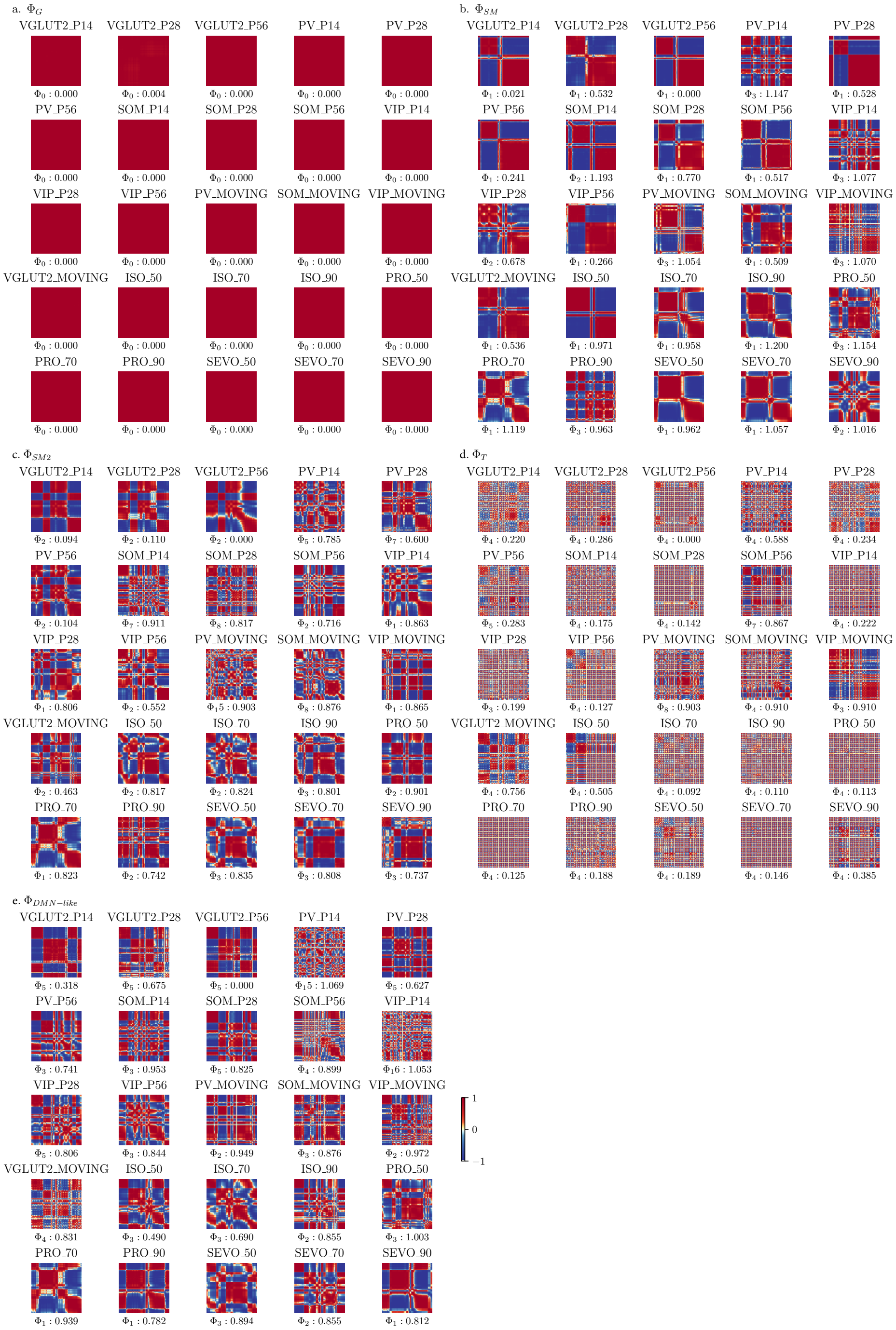

Supplementary Fig. 3: Correlation matrices of reconstructed time courses from standing/traveling waves. (a-e) Correlation matrices of reconstructed time courses from Φ_G, Φ_{SM1}, Φ_{SM2}, Φ_T and $\Phi_{DMN-like}$. We compared the correlation matrices of all waves from various cell types in different states with Φ_0, Φ_1, Φ_2, Φ_4 and Φ_5 from VGLUT2 at P56. We identified those with the highest similarity as belonging to the same category. The figure displays the correlation matrices of the waves with the highest similarities. The text below each matrix indicates the wave and its similarity to the corresponding wave in VGLUT2, measured by mean square error (MSE), with lower values indicating better similarity.

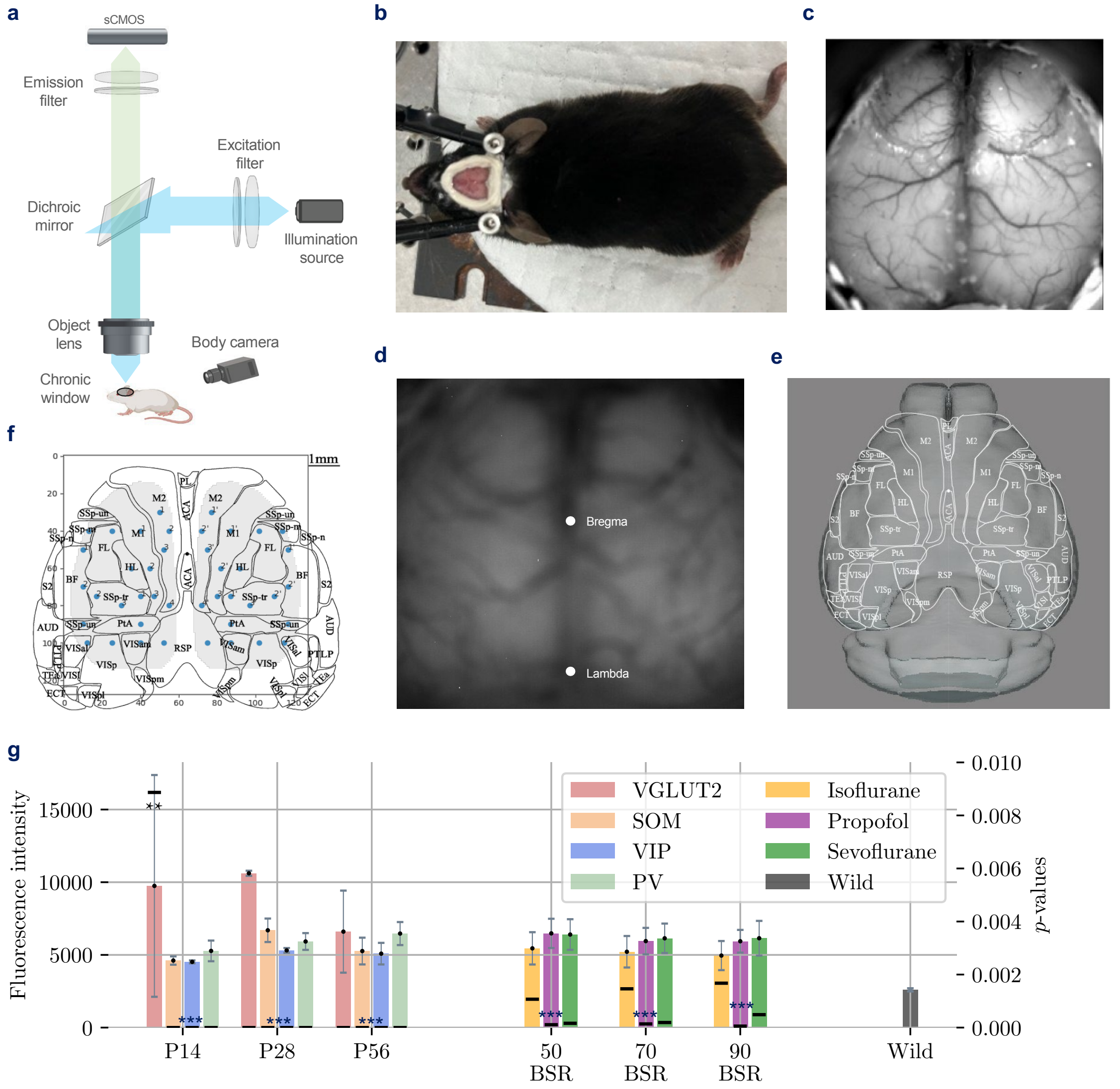

Supplementery Fig. 4: Fluorescence microscopy imaging in vivo and data processing. (a-b) Fluorescence imaging system. (c) The chronic through-bone window. (d) Fluorescent calcium signal, bregma and lambda are shown. (e) Brain region projections on the cortical surface based on the Allen Mouse Common Coordinate Framework v2 anatomy template. (f) Seed points selected for FC analysis. (g) Fluorescence intensity in different types of mice and conditions. The differences are come from sensitivity with each cell-type calcium reporters. (P14: 13 experiments over $n = 6$ VGLUT2 male mice. 17 experiments over $n = 6$ PV male mice. 16 experiments over $n = 16$ SOM male mice. 23 experiments over $n = 5$ VIP male mice. P28: 8 experiments over $n = 4$ VGLUT2 male mice. 32 experiments over $n = 7$ PV male mice. 19 experiments over $n = 6$ SOM male mice. 17 experiments over $n = 7$ VIP male mice. P56: 23 experiments over $n = 10$ VGLUT2 male mice. 17 experiments over $n = 13$ PV male mice. 20 experiments over $n = 10$ SOM male mice. 24 experiments over $n = 10$ VIP male mice. Anesthesia: $n = 7$ male mice examined over 9 independent experiments.)

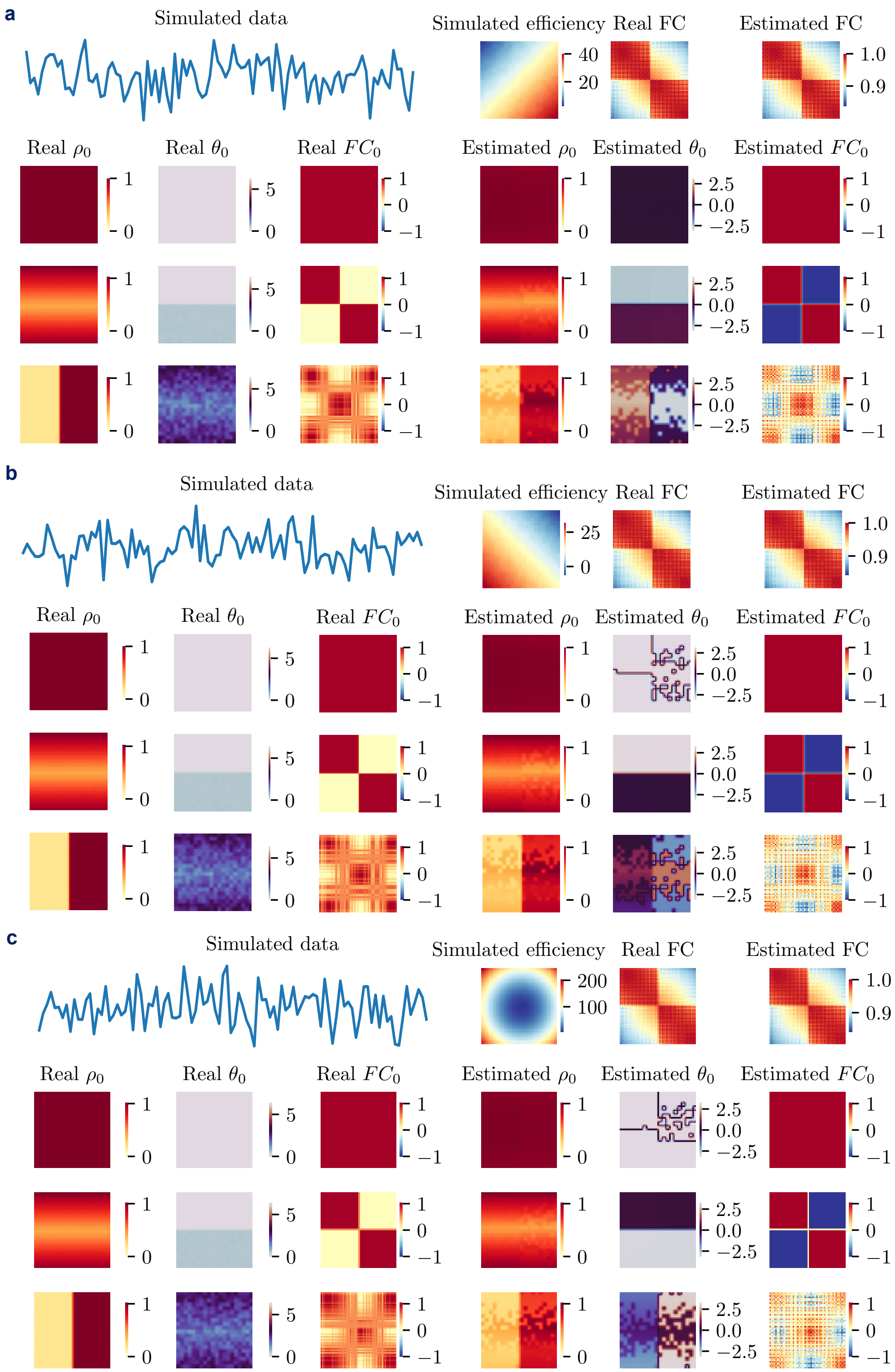

Supplementary Fig. 5: CPCA analysis using simulated data. (a-c) The results of three CPCA analyses using simulated data. In these three simulations, we simulated fluorescent proteins

with different efficiencies and of different spatial distributions, as shown in the figure of simulated efficiency.
