# Supplementary material for: Global spatiotemporal synchronizing structures of spontaneous neural activities in different cell types
Source: Nat Commun. 2024 Apr 3;15:2884. doi: 10.1038/s41467-024-46975-5 (PMC10991327; doi:10.1038/s41467-024-46975-5)
Supplement: Supplementary file 3 — Description of Additional Supplementary Files [file 41467_2024_46975_MOESM3_ESM.pdf]

Description of Additional Supplementary Files

Supplementary Movie 1: Spatiotemporal pattern of Φ0 in VGLUT2 at various awake and anesthesia state.
