# Supplementary material for: Global spatiotemporal synchronizing structures of spontaneous neural activities in different cell types
Source: Nat Commun. 2024 Apr 3;15:2884. doi: 10.1038/s41467-024-46975-5 (PMC10991327; doi:10.1038/s41467-024-46975-5)
Supplement: Supplementary file 5 — Reporting Summary [file 41467_2024_46975_MOESM5_ESM.pdf]

nature portfolio

Corresponding author(s): Pengcheng Li

Last updated by author(s): Aug 19, 2023

Reporting Summary

Nature Portfolio wishes to improve the reproducibility of the work that we publish. This form provides structure for consistency and transparency in reporting. For further information on Nature Portfolio policies, see our Editorial Policies and the Editorial Policy Checklist.

Statistics

For all statistical analyses, confirm that the following items are present in the figure legend, table legend, main text, or Methods section.

n/a	Confirmed	
☐	☒	The exact sample size (*n*) for each experimental group/condition, given as a discrete number and unit of measurement
☐	☒	A statement on whether measurements were taken from distinct samples or whether the same sample was measured repeatedly
☐	☒	The statistical test(s) used AND whether they are one- or two-sided<br>*Only common tests should be described solely by name; describe more complex techniques in the Methods section.*
☒	☐	A description of all covariates tested
☐	☒	A description of any assumptions or corrections, such as tests of normality and adjustment for multiple comparisons
☐	☒	A full description of the statistical parameters including central tendency (e.g. means) or other basic estimates (e.g. regression coefficient) AND variation (e.g. standard deviation) or associated estimates of uncertainty (e.g. confidence intervals)
☐	☒	For null hypothesis testing, the test statistic (e.g. *F*, *t*, *r*) with confidence intervals, effect sizes, degrees of freedom and *P* value noted<br>*Give P values as exact values whenever suitable.*
☒	☐	For Bayesian analysis, information on the choice of priors and Markov chain Monte Carlo settings
☒	☐	For hierarchical and complex designs, identification of the appropriate level for tests and full reporting of outcomes
☐	☒	Estimates of effect sizes (e.g. Cohen's *d*, Pearson's *r*), indicating how they were calculated

Our web collection on statistics for biologists contains articles on many of the points above.

Software and code

Policy information about availability of computer code

Data collection	No special software used in data collection.
Data analysis	All of our code used for this project is written in Python v3.10, making extensive use of Python packages, including NumPy v1.26.2, SciPy v1.9.1, statsmodels v0.13.5, matplotlib v3.8.2, and seaborn v0.12.2. Our custom code is provided at https://github.com/shih-liang/gssidnaim.

For manuscripts utilizing custom algorithms or software that are central to the research but not yet described in published literature, software must be made available to editors and reviewers. We strongly encourage code deposition in a community repository (e.g. GitHub). See the Nature Portfolio guidelines for submitting code & software for further information.

Data

Policy information about availability of data

All manuscripts must include a data availability statement. This statement should provide the following information, where applicable:

- Accession codes, unique identifiers, or web links for publicly available datasets
- A description of any restrictions on data availability
- For clinical datasets or third party data, please ensure that the statement adheres to our policy

We are currently organizing the data to improve data accessibility in preparation for public availability. They will be made available as soon as possible before publication. The data can also be obtained by contacting the corresponding author.

Research involving human participants, their data, or biological material

Policy information about studies with human participants or human data. See also policy information about sex, gender (identity/presentation), and sexual orientation and race, ethnicity and racism.

Reporting on sex and gender	No human samples were used in this study
Reporting on race, ethnicity, or other socially relevant groupings	No human samples were used in this study
Population characteristics	No human samples were used in this study
Recruitment	No human samples were used in this study
Ethics oversight	No human samples were used in this study

Note that full information on the approval of the study protocol must also be provided in the manuscript.

Field-specific reporting

Please select the one below that is the best fit for your research. If you are not sure, read the appropriate sections before making your selection.

☒ Life sciences ☐ Behavioural & social sciences ☐ Ecological, evolutionary & environmental sciences

For a reference copy of the document with all sections, see nature.com/documents/nr-reporting-summary-flat.pdf

Life sciences study design

All studies must disclose on these points even when the disclosure is negative.

Sample size	No statistical methods were used to pre-determine sample sizes. We imaged 6 VGLUT2 mice, 6 PV mice, 6 SOM mice, and 5 VIP mice at P14. At P28, we imaged 4 VGLUT2 mice, 7 PV mice, 6 SOM mice, and 7 VIP mice. At P56, we imaged 10 VGLUT2 mice, 13 PV mice, 10 SOM mice, and 10 VIP mice. We imaged 7 VGLUT2 mice under anesthesia at Adult. Our sample sizes are more than those reported in previous publications (Cabral et al Nat Comm 2023).
Data exclusions	For resting state analysis, data from periods with obvious body movement are excluded. For non-resting state analysis, only data from periods which mice are running on plate are included.
Replication	All FC data presented in this study were obtained from experimental replicates and validated through statistical analysis. For the analysis of resting state waves, we utilized CPCA on data from each individual mouse and compared the results at the group level. The waves extract from individual level are same with group level and their variance are shown in Fig 1,2,3 and 5.
Randomization	Mice were randomly assigned to age groups and resting-state/anesthesia states. During the anesthesia state, the depth of anesthesia in mice gradually increased, and data from the period of burst suppression ratio at 50%, 70%, and 90% are selected to analyze.
Blinding	Ages and anesthesia states cannot be concealed from the experimenters due to the evident differences in mouse size and condition. However, we made effort to ensure randomization in group allocation, consistent experimental procedures, data processing methods, and a consistent external environment for the experiments.

Reporting for specific materials, systems and methods

We require information from authors about some types of materials, experimental systems and methods used in many studies. Here, indicate whether each material, system or method listed is relevant to your study. If you are not sure if a list item applies to your research, read the appropriate section before selecting a response.

Materials & experimental systems

n/a	Involved in the study	
☒	☐	Antibodies
☒	☐	Eukaryotic cell lines
☒	☐	Palaeontology and archaeology
☐	☒	Animals and other organisms
☒	☐	Clinical data
☒	☐	Dual use research of concern
☒	☐	Plants

Methods

n/a	Involved in the study	
☒	☐	ChIP-seq
☒	☐	Flow cytometry
☒	☐	MRI-based neuroimaging

Animals and other research organisms

Policy information about studies involving animals; ARRIVE guidelines recommended for reporting animal research, and Sex and Gender in Research

Laboratory animals	We bred RCL-GCaMP6s mice64 (Ai96; B6.129S6-Gt(ROSA)26Sortm96(CAG-GCaMP6s)Hze/J, Jax #024106) with VGLUT2-ires-Cre mice (STOCK Slc17a6tm2(cre)Lowl/J, Jax #016963), PV-Cre mice (B6;129P2-Pvalbtm1(cre)Arbr/J, Jax #008069), SOM-IRES-Cre mice (STOCK Ssttm2.1(cre)Zjh/J, Jax #013044) and VIP-IRES-Cre mice (STOCK VIPtm1(cre)Zjh/J, Jax #010908), generating "VGLUT2-GCaMP6s" mice, "PV-GCaMP6s" mice, "SOM-GCaMP6s" mice, and "VIP-GCaMP6s" mice, respectively.
Wild animals	No wild animals were used in this study.
Reporting on sex	Results were analyzed from males. As our interest was development and state dependent patterns, we did not consider differences by sex.
Field-collected samples	No field-collected samples were used in this study.
Ethics oversight	All animal procedures were approved by the Hubei Provincial Animal Care and Use Committee and adhered to the experimental guidelines of the Animal Experimentation Ethics Committee of Huazhong University of Science and Technology in China.

Note that full information on the approval of the study protocol must also be provided in the manuscript.
